# Chromium Cycling in Redox‐Stratified Basins Challenges δ^53^Cr Paleoredox Proxy Applications

**DOI:** 10.1029/2022GL099154

**Published:** 2022-10-28

**Authors:** David J. Janssen, Jörg Rickli, Martin Wille, Oscar Sepúlveda Steiner, Hendrik Vogel, Olaf Dellwig, Jasmine S. Berg, Damien Bouffard, Mark A. Lever, Christel S. Hassler, Samuel L. Jaccard

**Affiliations:** ^1^ Institute of Geological Sciences University of Bern Bern Switzerland; ^2^ Oeschger Centre for Climate Change Research University of Bern Bern Switzerland; ^3^ Department Surface Waters Eawag: Swiss Federal Institute of Aquatic Science and Technology Kastanienbaum Switzerland; ^4^ Institute of Geochemistry and Petrology Department of Earth Sciences ETH Zurich Zurich Switzerland; ^5^ Marine Geology Leibniz Institute for Baltic Sea Research Rostock Germany; ^6^ Institute of Earth Surface Dynamics University of Lausanne Lausanne Switzerland; ^7^ Department of Environmental Systems Science ETH‐Zurich Zurich Switzerland; ^8^ Now at Marine Science Institute University of Texas at Austin TX Port Aransas USA; ^9^ Department F.‐A. Forel for Environmental and Aquatic Sciences University of Geneva Geneva Switzerland; ^10^ Institute of Earth Sciences University of Lausanne Lausanne Switzerland

**Keywords:** chromium, paleoproxy, stable isotopes, euxinia

## Abstract

Chromium stable isotope composition (δ^53^Cr) is a promising tracer for redox conditions throughout Earth's history; however, the geochemical controls of δ^53^Cr have not been assessed in modern redox‐stratified basins. We present new chromium (Cr) concentration and δ^53^Cr data in dissolved, sinking particulate, and sediment samples from the redox‐stratified Lake Cadagno (Switzerland), a modern Proterozoic ocean analog. These data demonstrate isotope fractionation during incomplete (non‐quantitative) reduction and removal of Cr above the chemocline, driving isotopically light Cr accumulation in euxinic deep waters. Sediment authigenic Cr is isotopically distinct from overlying waters but comparable to average continental crust. New and published data from other redox‐stratified basins show analogous patterns. This challenges assumptions from δ^53^Cr paleoredox applications that quantitative Cr reduction and removal limits isotope fractionation. Instead, fractionation from non‐quantitative Cr removal leads to sedimentary records offset from overlying waters and not reflecting high δ^53^Cr from oxidative continental weathering.

## Key Points

- Non‐quantitative Cr removal at the chemocline drives chromium (Cr) isotope fractionation and the accumulation of light Cr in euxinic deep waters
- Authigenic sediment δ^53^Cr differs from overlying water, contrary to paleoproxy application assumptions, and is similar to continental crust
- δ^53^Cr signals in these settings may therefore reflect internal redox processes and not fractionation due to oxidative subaerial weathering

## 1 Introduction

Authigenic chromium (Cr) enrichments and Cr stable isotope (δ^53^Cr) composition in sedimentary deposits are widely used to trace redox conditions throughout Earth's history (Frei et al., 2009; Planavsky et al., 2014; Reinhard et al., 2013; Wei et al., 2020). These applications, which interpret high δ^53^Cr to signal elevated oxidative weathering of the continents, are based on the redox control of Cr solubility and stable isotope fractionation in aqueous environments. Reduced Cr (Cr(III)) is particle reactive and can be readily removed from the water column (Richard & Bourg, 1991), while oxidized Cr(VI) is soluble, with isotope fractionation resulting in enrichments of isotopically light Cr in Cr(III) (Ellis et al., 2002; Wanner & Sonnenthal, 2013). As a consequence of Cr reduction and removal, natural systems with strong O_2_ deficiency are associated with dissolved Cr depletions as well as elevated dissolved δ^53^Cr (Huang et al., 2021; Moos et al., 2020; Murray et al., 1983; Nasemann et al., 2020; Rue et al., 1997).

Paleo‐reconstructions using sedimentary δ^53^Cr records apply the assumption that Cr is efficiently sequestered into sediment phases under reducing conditions (anoxia or euxinia). Thus, complete (i.e., quantitative) Cr redox conversion prevents isotopic fractionation, resulting in sediment‐hosted authigenic δ^53^Cr equivalent to the overlying water column (Frei et al., 2009, 2020; Reinhard et al., 2013, 2014; Wei et al., 2020). However, the few studies of euxinic waters so far indicate the opposite, that is, enrichment rather than removal of dissolved Cr(III) (Achterberg et al., 1997; Davidson et al., 2020; Emerson et al., 1979), and no data from co‐localized euxinic waters and sediments are available. Due to the lack of data on Cr partitioning across redox interfaces and into underlying sediments, isotopic offsets resulting from partial (i.e., non‐quantitative) Cr(VI) reduction during removal to sediments have been neglected in δ^53^Cr paleoproxy interpretations.

**Figure 1:**
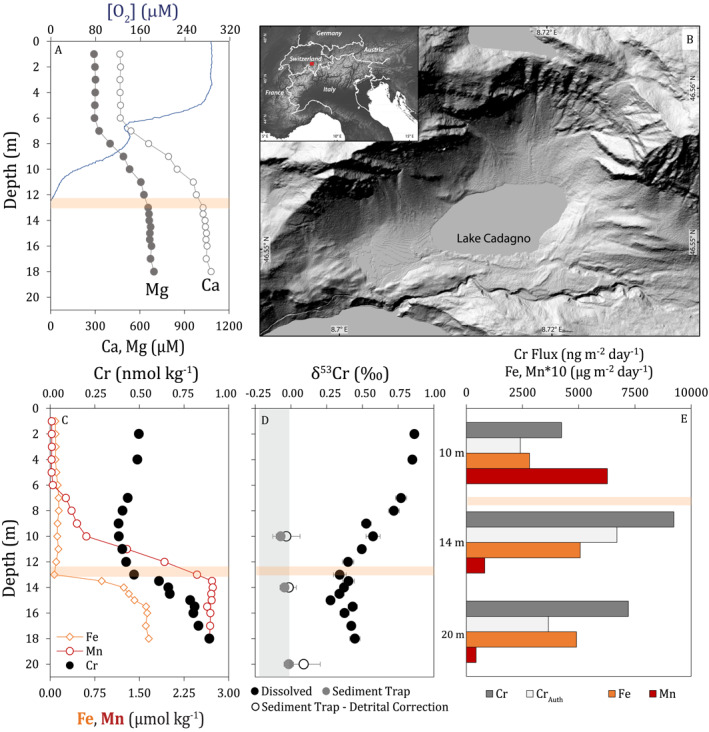
Water column data. (a): [O_2_], [Ca] and [Mg]. (b): map showing the location of Lake Cadagno. (c): dissolved [Cr], [Fe] and [Mn]. (d): dissolved and sediment trap δ^53^Cr (uncorrected and corrected for detrital chromium (Cr), see Section S.5 in Supporting Information S1). (e): Sediment trap Cr, Fe and Mn fluxes (note different depth range). The orange box in (a and c–e) indicates the chemocline. The gray box in (d) indicates average detrital δ^53^Cr (Schoenberg et al., 2008).

Redox‐stratified basins are valuable settings to build the geochemical understandings necessary for developing and interpreting paleoproxies for the early stratified oceans. One such system, the meromictic alpine Lake Cadagno (Switzerland) (Figures 1a and 1b), has been used extensively as an analog for the Phanerozoic and Proterozoic oceans (Canfield et al., 2010; Dahl et al., 2010; Ellwood et al., 2019; Xiong et al., 2019) due to the intermediate sulfate levels and sulfidic bottom waters supporting anoxygenic phototrophs in the chemocline (Tonolla et al., 2003). To mechanistically constrain Cr cycling across redox gradients and incorporate these into the Cr‐based paleoproxy framework, we present [Cr] and δ^53^Cr in the water column (total dissolved) and sediments (near‐total digests and leachates), along with sinking particulates. These observations question fundamental assumptions inherent to the δ^53^Cr paleoproxy applications and will help to inform future δ^53^Cr‐based paleo‐reconstructions.

## 2 Study Area and Methods

### 2.1 Study Area

Lake Cadagno, at 1921 m elevation, is a 21 m deep meromictic alpine lake in Switzerland characterized by a permanent chemocline near 13 m depth. The initially oxic lake formed ∼13,500 YBP. A transition phase persisted between 10,00 and 9,000 YBP, followed by euxinic monimolimnion conditions, which have been generally stable until present (Berg et al., 2022). The lake's distinct geochemistry and microbial communities have been well‐characterized. Waters below the chemocline are fed by groundwater input from a karstic system composed of dolomite and gypsum, and are therefore rich in Ca^2+^, Mg^2+^, SO_4_^2−^ and HCO_3_^−^ relative to overlying waters (Del Don et al., 2001, Figure 1). In anoxic deep waters, dissolved concentrations reach 10^2^ μmol kg^−1^ sulfide, ∼3.5 mmol kg^−1^ sulfate, and ∼1 μmol kg^−1^ Fe (e.g., Ellwood et al., 2019, see supplement). High densities of anoxygenic photoautotrophic green and purple sulfur bacteria are found at the chemocline in summer (Tonolla et al., 2003). These bacteria exert control on geochemical gradients in this zone (e.g., S, Fe; Del Don et al., 2001; Berg et al., 2016) and can form a 0.3–1.2 m thick mixed layer through bioconvection during summer (Sepúlveda Steiner et al., 2019, 2021; Sommer et al., 2017).

### 2.2 Sampling, Sample Purification and Analysis

Dissolved Lake Cadagno water samples were collected on 28–29 August 2017 from a floating platform at the deepest part of the lake. Sediment traps were deployed on 10 July 2017 and recovered on 6 September 2017. Sediments were sampled in summer 2019 and summer 2020 (Berg et al., 2022), freeze dried, and hand milled with an agate mortar and pestle. Baltic Sea samples were collected in the central Landsort Deep (site LD1; 435 m water depth, Häusler et al., 2018) onboard RV Poseidon (POS507, 29 October 2016).

**Figure 2:**
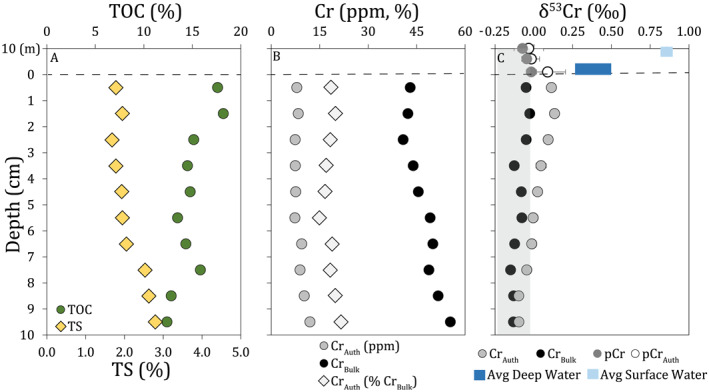
Near‐surface sediment data. (a): bulk sediment parameters (TOC, TS). (b): near‐total digest (Cr_Bulk_) and leach (Cr_Auth_) concentrations, and Cr_Auth_ as % Cr_Bulk_. (c): δ^53^Cr_Bulk_ and δ^53^Cr_Auth_ with average surface and deep water dissolved δ^53^Cr and sediment trap δ^53^Cr (total and detrital‐corrected), along with average detrital δ^53^Cr (gray box).

Water column sampling and spiking (with a ^50^Cr‐^54^Cr double spike) followed standard procedures, and are discussed in Section S.1 in Supporting Information S1. Samples were processed through three stages of column chromatography: (i) Fe removal using AG1‐X8 resin in 6.4 M HCl (Scheiderich et al., 2015), followed by (ii) anion and (iii) cation chromatography as described elsewhere (Janssen et al., 2020; Nasemann et al., 2020; Rickli et al., 2019). Sediment near‐total digests were prepared using inverse aqua regia with H_2_O_2_. Authigenic sediment phases (primarily organic matter, potentially also sulfides, Figure 2a) were targeted with a 30% v/v H_2_O_2_ leach at pH = 2 following Rauret et al. (1999) (see Section S.1 in Supporting Information S1). Leach and digest subsamples were spiked with a ^50^Cr‐^54^Cr double spike, dried and processed through steps (i) and (iii). Reagents were either sub‐boiling distilled (acids) or Romil UpA and Fisher Optima grade (H_2_O_2_). Ancillary data are described in the supplement.

Sediment trap fluxes are calculated with and without corrections for lithogenic contributions (Figures 1 and 2, Table 1). Uncertainties on corrected data (pCr_Auth_) follow standard error propagation using lithogenic Cr/Al (Rudnick & Gao, 2014) and δ^53^Cr = −0.12 ± 0.1 ‰ (Schoenberg et al., 2008). Such corrections, which rely on normalizations to major crustal elements considered minimally mobile (e.g., Al), are complicated by the authigenic components of these elements in Lake Cadagno (Figures S6 and S7 in Supporting Information S1). Therefore, these corrections underestimate pCr_Auth_ and overestimate δ^53^Cr_Auth_, with true authigenic values lying between the corrected and uncorrected data (See Section S.5 in Supporting Information S1). Detrital corrections were not applied to sediment leach data as chemical leaches were more gentle than sediment trap digests (See Section S.1.2 in Supporting Information S1) enhancing the impact of authigenic phases of the normalizing element and exacerbating artifacts from improper corrections (Figures S6 and S7 in Supporting Information S1).

**Table 1:** Sediment Trap, Diffusive and Burial Fluxes

	Depth	pCr				pCr_Auth_			
Downward fluxes	m	ng Cr	ng Cr m^−2^ day^−1^	δ^53^Cr	2SEM	ng Cr	ng Cr m^−2^ day^−1^	δ^53^Cr	2SEM
Sediment Trap	10	3.2 × 10^3^	4.2 × 10^3^	−0.07	0.03	1.8 × 10^3^	2.4 × 10^3^	−0.03	0.09
Sediment Trap	14	7.0 × 10^3^	9.2 × 10^3^	−0.05	0.03	5.1 × 10^3^	**6.7** × **10** ^**3**^	−0.02	0.05
Sediment Trap	20	5.4 × 10^3^	7.2 × 10^3^	−0.02	0.03	2.8 × 10^3^	3.6 × 10^3^	0.09	0.11
	Euxinic zone [Cr]	Chemocline base [Cr]	Δ[Cr]	ΔZ	K	Flux	Flux		
Upward Fluxes	nmol kg⁻^1^	nmol kg⁻^1^	nmol cm⁻³	cm	cm^2^ s^‒1^	nmol cm⁻^2^ s⁻^1^	ng m⁻^2^ d⁻^1^		
Turbulent Diffusion	0.67	0.47	0.0002	150	1.1 × 10^‒1^	1.4 × 10^−7^	**6.4** × **10** ^**3**^		
Molecular Diffusion	0.67	0.47	0.0002	150	3.4 × 10^‒6^	4.6 × 10^−12^	2.7 × 10^−1^		
	Sediment Acc. Rate^1^	Wet Sed. Density (ρ)	Porosity (β)	Cr_Auth_	F_Burial_	F_Burial_	F_Burial_		
Cr burial	mm yr^−1^	g cm^−3^	g H_2_O g wet sed.^−1^	ppm	nmol cm^‒2^ y^‒1^	ng cm^−2^ y^−1^	ng m^−2^ d^−1^		
Authigenic Cr Burial	4–6	2.30	0.33	8	0.9–1.4	49–74	1.3–2.0 × 10^3^		

*Note.* Fluxes to and from the chemocline are in bold. Sediment trap data include both total (pCr) and detrital‐corrected (pCr_Auth_) fluxes (See Section S.5 in Supporting Information S1). Upward turbulent diffusive fluxes of dissolved [Cr] are based on [Cr] gradients from the chemocline to 1.5 m below the chemocline. ^1^Birch et al., 1996. See Section S.3.3 in Supporting Information S1 for further details on authigenic burial estimates.

Purified δ^53^Cr samples were dissolved in 0.7–1 mL 0.5 M HNO_3_ and analyzed with a Neptune Plus MC‐ICP‐MS (ThermoFisher) (Rickli et al., 2019). Internal sample uncertainty (2 SEM) and session reproducibility from NIST standards (*n* ≈ 10, 2 SD) is typically around 0.02–0.03 ‰. External uncertainty has previously been estimated at ±0.033 ‰ for full sample replicates (Janssen et al., 2020). Pure standard reference materials (Merck Cr(III) standard, this study: δ^53^Cr = −0.45 ± 0.03 ‰, *n* = 10; δ^53^Cr = −0.44 ± 0.02 ‰, Schoenberg et al., 2008) and USGS reference materials (Table S3 in Supporting Information S1) agree with published values.

## 3 Lake Cadagno Results and Discussion

### 3.1 Water Column

Dissolved [Cr] is stable in the surface mixed layer ([Cr] = 0.5 nmol kg^−1^, Figure 1c). A broad [Cr] minimum is found at 8–11 m depth, above the chemocline (∼12–13 m in summer 2017, Figure S1 in Supporting Information S1; Sepúlveda Steiner et al., 2021). Particulate Fe and Mn oxides form within and above the chemocline in Lake Cadagno, driven by upward transport of dissolved Fe(II)—a known Cr reductant (Richard & Bourg, 1991; Wanner & Sonnenthal, 2013)—and Mn(II) from the anoxic zone (Ellwood et al., 2019). As these oxides sink below the chemocline, they are reductively dissolved within the anoxic zone, and particulate Fe sulfides are formed (Ellwood et al., 2019). The [Cr] minimum lies within the most stratified portion of the water column (Figure S2 in Supporting Information S1) and corresponds to depths with enhanced formation and sinking of Fe and Mn oxides (see figure 5 in Ellwood et al., 2019). Therefore the [Cr] minimum is mechanistically consistent with Cr reduction coupled to Fe oxidation followed by Cr scavenging onto metal oxides.

Chromium concentrations begin increasing just above the chemocline, exceeding surface concentrations immediately below the chemocline. Dissolved [Fe] and [Mn] also increase over this range, with the [Cr] increase more closely mirroring [Fe] (Figure 1c). Matching [Cr], δ^53^Cr is stable in the surface mixed layer (δ^53^Cr = +0.86 ‰) (Figure 1d), with the isotopically heavy dissolved Cr signal likely reflecting surface water input to the lake, consistent with expectations from oxidative terrestrial weathering and global observations of riverine δ^53^Cr (Wei et al., 2020). δ^53^Cr decreases with depth, indicating the accumulation of isotopically light Cr in deep waters following Cr release during metal oxide reduction.

Sinking fluxes of authigenic particulate Cr (pCr_Auth_) increase near the chemocline, with maximum fluxes observed in the 14 m trap. Exported pCr is isotopically lighter than dissolved Cr, with Δ^53^Cr_patriculate‐dissolved_ ≈ −0.6 ± 0.1 ‰ near the chemocline. This is comparable to net fractionations observed in other natural systems, but is much lower than theoretical values observed in lab studies (e.g., Wanner & Sonnenthal, 2013), which likely reflects incomplete removal of reduced Cr resulting in lower apparent fractionation factors for Cr reduction and removal (Huang et al., 2021; Nasemann et al., 2020; Wang, 2021). Isotopically, pCr_Auth_ is indistinguishable from the lithogenic background (δ^53^Cr = −0.12 ± 0.10 ‰, Schoenberg et al., 2008). Export fluxes of pCr_Auth_ and pMn decrease with depth in the euxinic zone (Figures 1e and Table 1; Table S2 in Supporting Information S1), while pFe species shift from oxide‐dominated above the chemocline to sulfides below (Berg et al., 2022; Ellwood et al., 2019), suggesting an Fe‐Mn‐oxyhydroxide shuttle and resulting in much of the Cr exported across the chemocline being released before the deepest sediment trap (20 m). The behavior of pCr is thus consistent with dissolved data indicating Cr release and accumulation in the euxinic zone following reduction of Fe and Mn oxides. Despite depth‐dependent variability in pCr_Auth_, exported δ^53^Cr is uniform within analytical uncertainty, indicating no fractionation during Cr release.

Deep layers of euxinic water bodies are assumed to remain quiescent given the strong stratification, and therefore the vertical gradient of [Cr] should be smoothed by an upward molecular diffusive flux. However, the pCr_Auth_ export (∼6.7 × 10^3^ ng Cr m^−2^ day^−1^) is orders of magnitude larger than the molecular flux of dissolved Cr (Table 1, Section S.3 in Supporting Information S1) while the observed [Cr] depletion is subtle. Despite the apparent quiescence of the deep interiors of lakes, various physical processes maintain a moderate and intermittent energetic structure (Saggio & Imberger, 2001), including in Lake Cadagno (Wüest, 1994). Here our concurrent microstructure observations indicate that vertical fluxes are sustained by turbulent diffusivity (K_oc_, Osborn & Cox, 1972), with a mean K_oc_ in the layer of interest of 10^−1^ cm^2^ s⁻^1^ (Figure S2 in Supporting Information S1; four orders of magnitude larger than molecular diffusivity). This results in upward turbulent flux estimates comparable to sinking pCr fluxes, while the authigenic burial flux is comparatively smaller (∼25% of turbulent and particulate fluxes, Table 1). The background turbulence thereby explains Cr profiles and, particularly, the lack of a pronounced [Cr] minimum and local δ^53^Cr maximum above the chemocline, due to the substantial upward turbulent transport of isotopically light Cr. The result is similar to “cryptic” cycles, where rapid and localized redox cycling masks the expected biogeochemical signals (e.g., Berg et al., 2016), though rather as a larger‐scale physical‐geochemical transport cycle.

Observed deep water [Cr] enrichments likely reflect the integrated accumulation of Cr released from sinking particles and near‐surface sediments as well as the potential contribution from groundwater (Del Don et al., 2001), with concentrations further modified by turbulent mixing. As there is no modification of particulate δ^53^Cr with depth, released Cr must be of similar isotope composition as the particulates (δ^53^Cr ≈ 0 ‰). Therefore, any groundwater Cr must be isotopically heavier than 0 ‰ (see Section S.4 in Supporting Information S1). However, while subaquatic springs likely influence specific aspects of Lake Cadagno deep water [Cr] and δ^53^Cr, other euxinic basins exhibit similar large‐scale [Cr] and δ^53^Cr depth‐trends (see below). Therefore, a shared set of geochemical and physical controls likely shape the common large‐scale [Cr] and δ^53^Cr trends across diverse euxinic basins.

### 3.2 Sediments

Authigenic Cr (Cr_Auth_) in near‐surface sediments is isotopically lighter than deep water, with similar δ^53^Cr as sinking particles (Figure 2). We do not observe strong [Cr_Auth_] enrichments relative to [Cr_Bulk_], despite stably euxinic deep waters and large Cr fluxes from the chemocline, and Cr_Auth_ burial fluxes are small relative to chemocline particulate fluxes, reflecting significant Cr release from sinking particles (Table 1, Section S.3.3 in Supporting Information S1). Sediment Mn and Fe(III) content is low relative to sinking particles (Figure 1, Ellwood et al., 2019; Berg et al., 2022), supporting a Fe‐Mn‐oxyhydroxide shuttle for Cr, with Cr release following oxide dissolution. Bulk sediment δ^53^Cr (δ^53^Cr_Bulk_) is indistinguishable from average continental crust (−0.12 ± 0.10 ‰, Schoenberg et al., 2008) at all depths. δ^53^Cr_Auth_ is slightly higher than crustal signatures in the uppermost sediments; however, δ^53^Cr_Auth_ decreases with sediment depth and is indistinguishable from this inventory below 5 cm. δ^53^Cr_Bulk_ and δ^53^Cr_Auth_ converge in the upper 10 cm of the sediment, suggesting modification of δ^53^Cr through early diagenesis (sedimentation rates ∼4–6 mm yr^−1^, Birch et al., 1996), similar to reports of Cr homogenization in black shales (Frank, et al., 2020).

While surface sediment δ^53^Cr_Auth_ equals sinking particulate δ^53^Cr, non‐quantitative water column Cr removal results in isotope fractionation and an offset between sediments and dissolved δ^53^Cr. This fractionation, along with potential early diagenetic modification, leads coincidentally to a δ^53^Cr_Auth_ equivalent to detrital reservoirs (Figure 2). δ^53^Cr_Auth_ is approximately 0.6 ‰ lower than dissolved δ^53^Cr in Lake Cadagno at the [Cr] minimum; however, variable dissolved δ^53^Cr results in inconsistent offsets between δ^53^Cr_Auth_ and surface, deep and chemocline dissolved δ^53^Cr. [Cr_Bulk_] and δ^53^Cr_Bulk_ in sapropel samples, obtained from a sediment core spanning the last ∼12 ka (Berg et al., 2022) remain relatively stable with depth. δ^53^Cr_Bulk_ is indistinguishable from lithogenic background and δ^53^Cr_Auth_ is indistinguishable from or only slightly heavier than lithogenic background (Figure S5 in Supporting Information S1). Thus, despite deposition in an anoxic basin below an oxidizing atmosphere, sediment δ^53^Cr_Bulk_ and δ^53^Cr_Auth_ provide no isotopically heavy Cr record (reflecting oxidative weathering and high dissolved δ^53^Cr). These data imply that in other redox‐stratified settings, an isotopically heavy dissolved δ^53^Cr pool does not necessarily result in isotopically heavy δ^53^Cr_Auth_ or δ^53^Cr_Bulk_ records, and that constraints on water column fractionation are needed to reconstruct dissolved δ^53^Cr of overlying waters and oxidative weathering processes from sedimentary δ^53^Cr records.

## 4 Synthesis of Redox‐Stratified Systems

**Figure 3:**
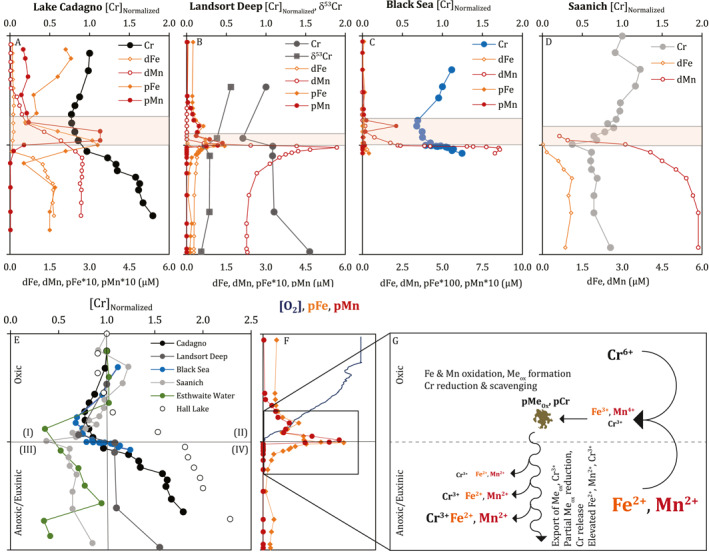
Compilation of anoxic basin data with conceptual mechanistic diagram. Chromium, Fe (orange) and Mn (red) concentrations in the dissolved (open circles, dFe and dMn) and particulate (filled circles, pFe and pMn) phases from Lake Cadagno ((a), black, this study, pFe and pMn: Ellwood et al., 2019), Landsort Deep ((b), dark gray, this study), the Black Sea ((c), blue, Cr: Mugo, 1997, Fe and Mn: Lewis & Landing, 1991), and Saanich Inlet ((d), Emerson et al., 1979, light gray, only dissolved data). (e) combines (a–d) with Esthwaite Water (green, Achterberg et al., 1997) and Hall Lake (white, Balistrieri et al., 1994). In (a)–(f), the *y*‐axis ranges from the surface to sediments, with the chemocline at the center. In (a–e), [Cr] is normalized to surface concentrations. The orange shading in (a–d) indicates the zone of enhanced Fe and Mn redox cycling above the chemocline. (f–g) show a schematic of metal cycling in this zone, using Landsort Deep data (f) (see Table S8 in Supporting Information S1).

δ^53^Cr has received significant attention as a paleoproxy for O_2_ availability, especially in the Proterozoic. While Lake Cadagno is a promising analogue for biogeochemical cycling in the Proterozoic ocean, it remains a small Alpine lake with unique physical and biogeochemical cycling. To assess the relevance of these data to other modern redox‐stratified systems and to oceanic systems throughout geologic time, we compiled new dissolved [Cr] and δ^53^Cr data from the weakly euxinic Landsort Deep site in the Baltic Sea (Häusler et al., 2018), together with published [Cr] data from other redox‐stratified basins (Figure 3).

In the Landsort Deep, Cr is removed near the chemocline (Figure 3b, Table S7 in Supporting Information S1), coincident with a maximum in particulate Fe and Mn oxides. High dissolved [Cr] and low δ^53^Cr are found in underlying waters where dissolved [Fe] and [Mn] reach μM levels, indicating an accumulation of isotopically light Cr in euxinic waters as in Lake Cadagno. These data support isotope fractionation during non‐quantitative Cr reduction and removal via scavenging onto metal oxides slightly above the chemocline, with Cr release following oxide reduction in euxinic waters and/or surface sediments.

Data from other redox‐stratified basins are similar, despite wide ranges in dissolved H_2_S (10°–10^2^ μmol kg^−1^) and Fe (10^−2^–10^2^ μmol kg^−1^) concentrations and Fe/H_2_S (10^−4^–10^3^) (Table S9 in Supporting Information S1). This includes Lake Cadagno and the Landsort Deep, Saanich Inlet (Emerson et al., 1979, BC Canada, see also Davidson et al., 2020), the Black Sea (Mugo, 1997), Esthwaite Water (UK, Achterberg et al., 1997) and Hall Lake (Wa USA; Balistrieri et al., 1994) (Figures 3a–3e). These can be more directly compared by normalizing profiles to [Cr]_surface_ and relative depth above and below the chemocline, creating four quadrants: (I) [Cr] < [Cr]_surface_ in oxic waters, (II) [Cr] > [Cr]_surface_ in oxic waters, (III) [Cr] < [Cr]_surface_ in anoxic waters, and (IV) [Cr] > [Cr]_surface_ in anoxic waters (Figure 3e). All environments lie within (I) slightly above the chemocline, indicating non‐quantitative Cr removal. Anoxic deep waters fall into quadrant (IV) in most settings, indicating Cr accumulation rather than removal. Basins with only seasonal anoxia (Saanich Inlet, Esthwaite Water) differ, showing a slight increase in [Cr]_Normalized_ just above the chemocline but remaining in zone (III) at depth. This may indicate insufficient time to allow Cr accumulation through the Fe‐Mn shuttle, due to their only seasonally anoxic nature.

This compilation from diverse permanently and seasonally anoxic systems confirms the general trends observed in Lake Cadagno—(i) non‐quantitative Cr removal above the chemocline (∼20–60% of [Cr]_surface_), suggesting isotope fractionation and the transport of low δ^53^Cr to anoxic waters, and (ii) Cr accumulation in anoxic deep waters indicating poor sequestration of Cr into sediments. Therefore, reconstructions of water column or weathering δ^53^Cr signals from sediments deposited in these environments require accounting for fractionation during Cr removal as well as internal cycling resulting in variable water column δ^53^Cr.

Chromium depletions consistently occur above the chemocline, coincident with elevated dissolved and particulate [Fe] and [Mn] (orange box in Figures 3a–3d). The sharpness of the Cr depletion above and subsequent increase below the chemocline can be explained by natural variability in the systems (see also Dellwig et al., 2012), including absolute depth ranges (10–10^3^ m), the relative width of the Fe‐Mn redox zone (orange box height, Figures 3a–3d) and upward diffusive transport. Shallower systems (e.g., Hall and Cadagno Lakes), thicker Fe‐Mn redox zones, and elevated diffusivity correspond with broader Cr minima and more gradual Cr increases below the chemocline.

In agreement with previous studies (e.g., Balistrieri et al., 1994; Mugo, 1997), the consistent Cr minimum in the zone of intense Fe‐Mn redox cycling supports the widespread control of these metals on Cr removal in anoxic systems, whereby Cr reduction coupled to Fe oxidation and subsequent Cr(III) scavenging on particulate metal oxides drives Cr removal (Figures 3f and 3g), followed by oxide reduction and Cr release in the anoxic zone or near the sediment surface. The broadly similar Cr trends across these diverse freshwater and marine systems indicates that our data from Cadagno are generally relevant to other systems and can be used to revise the interpretational framework of δ^53^Cr paleoproxy applications. Specifically, these data indicate that:

1. Cr is partially removed above the chemocline, but is not efficiently removed from the water column in anoxic systems, both at and below the chemocline
2. Dissolved Cr generally accumulates in anoxic deep water
3. Sediments from redox‐stratified basins may not always show high [Cr_Auth_] enrichments
4. Reconstructing water column δ^53^Cr from δ^53^Cr_Auth_ within these settings requires accounting for variable water column δ^53^Cr, fractionation during Cr removal and early diagenesis
5. δ^53^Cr_Auth_ within these settings may therefore not directly reflect Cr fractionation originating from oxidative subaerial weathering

## 5 Conclusions and Implications

Available data across a range of redox‐stratified marine and lacustrine settings share fundamental features—namely, local [Cr] minima with non‐quantitative Cr removal slightly above the chemocline, and increasing [Cr] below—with permanently anoxic basins showing deep water dissolved Cr accumulation rather than efficient removal. Given the isotope fractionation associated with Cr reduction, non‐quantitative removal suggests that sedimentary authigenic δ^53^Cr should not match the water column, contrasting previous assumptions (e.g., Frei et al., 2009, 2020; Reinhard et al., 2013, 2014; Wei et al., 2020). Instead, sediment δ^53^Cr_Auth_ is isotopically offset from the water column, a factor that must be considered for paleoreconstructions. Furthermore, variable water column δ^53^Cr, as well as potential early diagenetic modification of δ^53^Cr_Auth_, suggests there is no consistent offset between δ^53^Cr_Auth_ and water column δ^53^Cr. Indeed, our Lake Cadagno data show that sinking particulate δ^53^Cr is isotopically comparable to sediment δ^53^Cr_Auth_, while dissolved δ^53^Cr decreases from surface waters to euxinic deep waters, and therefore dissolved δ^53^Cr differs from δ^53^Cr_Auth_ by variable extents.

Despite a strongly oxidizing atmosphere throughout the entire history of Lake Cadagno, we find no significantly fractionated sediment δ^53^Cr_Auth_. Evidently, oxidative weathering does not necessarily result in high δ^53^Cr_Auth_. To the contrary, the relatively small ranges of dissolved δ^53^Cr throughout modern systems (surface waters ≈ +0.2 to +1.2 ‰, Wei et al., 2020; Horner et al., 2021) coupled with effective fractionation for the reduction and removal of Cr (Δ^53^Cr ≈ −0.4 to −1.3 ‰; this study; Janssen et al., 2020, 2021; Moos et al., 2020; Nasemann et al., 2020; Huang et al., 2021; Wang, 2021) indicate that authigenic sedimentary δ^53^Cr records could easily be comparable to unfractionated continental crust (δ^53^Cr = −0.12 ± 0.10 ‰, Schoenberg et al., 2008). In other words, Cr removal from a water column inventory of +0.2 to +1.2 ‰, with a Δ^53^Cr_particle‐dissolved_ of −0.4 to −1.3 ‰ is expected to yield an authigenic sedimentary δ^53^Cr that is, at times, within the range of −0.2 to 0.0 ‰. Consequently, caution should be used in the interpretation of δ^53^Cr records from redox‐stratified basins, which reflect local redox‐related fractionation processes superimposed on oxidative subaerial weathering signals.

## Supporting information

Supporting Information S1

## Data Availability

Data are presented in the Supporting Information S1 and Table 1. Vertical microstructure data for turbulent diffusion estimations is available at https://doi.org/10.5281/zenodo.3507638. Chemical and CTD data are also available in the following open access datasets in the Zenodo repository: http://doi.org/10.5281/zenodo.7125831 and http://doi.org/10.5281/zenodo.7127882, respectively.
