# Supplementary material for: Chromium Cycling in Redox‐Stratified Basins Challenges δ53Cr Paleoredox Proxy Applications
Source: Geophys Res Lett. 2022 Oct 28;49(21):e2022GL099154. doi: 10.1029/2022GL099154 (PMC9787902; doi:10.1029/2022GL099154)
Supplement: Supplementary file 1 — Supporting Information S1 [file GRL-49-e2022GL099154-s001.pdf]

Geophysical Research Letters

Supporting Information for

Chromium cycling in euxinic basins challenges δ^{53}Cr paleoredox proxy applications

David J. Janssen[a,b,c], Jörg Rickli[a,b,d], Martin Wille[a], Oscar Sepúlveda Steiner[c], Hendrik Vogel[a,b], Olaf Dellwig[e], Jasmine S. Berg[f], Damien Bouffard[c], Mark A. Lever[g], Christel S. Hassler[h], Samuel L. Jaccard[a,b,i]

[a]Institute of Geological Sciences, University of Bern, Baltzerstrasse 1-3, 3012 Bern Switzerland.
[b]Oeschger Centre for Climate Change Research, University of Bern, Hochschulstrasse 4, 3012 Bern Switzerland.
[c]Department Surface Waters, Eawag: Swiss Federal Institute of Aquatic Science and Technology, Seestrasse 79, 6047 Kastanienbaum Switzerland.
[d]Institute of Geochemistry and Petrology, Department of Earth Sciences, ETH Zurich, Sonneggstrasse 5, 8092 Zurich, Switzerland.
[e]Marine Geology, Leibniz Institute for Baltic Sea Research, Seestrasse 15, 18119 Rostock, Germany.
[f]Institute of Earth Surface Dynamics, University of Lausanne, Geopolis, 1015 Lausanne, Switzerland
[g]Department of Environmental Systems Science, ETH-Zurich, Zurich, Switzerland
[h]Department F.-A. Forel for Environmental and Aquatic Sciences, University of Geneva, Switzerland.
[i]Institute of Earth Sciences, University of Lausanne, Geopolis, 1015 Lausanne, Switzerland.

Contents of this file

Text sections
S.1 Lake Cadagno background, sampling and methodology
S.2 Mixing in Lake Cadagno, August 2017
S.3 Chromium fluxes in Lake Cadagno
S.4 Particulate oxide shuttles and groundwater
S.5 Discussion of authigenic corrections
S.6 Landsort Deep (Baltic Sea) data
S.7 Compilation of literature data

Figures
Figure S1: Dissolved oxygen (a) and turbidity (b) in Lake Cadagno during summer 2017
Figure S2: Turbulent diffusivity from July and August 2017
Figure S3: Chromium, Fe, P, Mn and Mg bi-variant plots
Figure S4: Cr-δ^{53}Cr plot
Figure S5: Full sediment core [Cr] and δ^{53}Cr data from Lake Cadagno
Figure S6: Distributions of dissolved Al in Lake Cadagno
Figure S7: Relationships between Al and other parameters in short core (0-10 cm) sediment leaches
Figure S8: Comparison of original leachate data and authigenic Al corrections

Figure S9: Landsort Deep (Baltic Sea) water column profiles

Tables

Table S1: Water column data
Table S2: Sediment trap data
Table S3: Sediment standards
Table S4: Sediment leaches
Table S5: Sediment near-total digests
Table S6: Wet and dry sediment mass
Table S7: Landsort Deep (Baltic Sea) water column Cr data
Table S8: Landsort Deep (Baltic Sea) water column Fe and Mn data
Table S9: Sulfide, Fe and Fe/H_2S ratios in redox-stratified systems included in Figure 3

Introduction

Supplementary figures, discussion, methodological details, and tables, including full metal data.

S.1 Lake Cadagno background, sampling and methodology

S.1.1 Biogeochemical background for Lake Cadagno

Lake Cadagno is a 21 m deep meromictic alpine lake in Ticino, Switzerland, with a persistently anoxic subsurface separated from oxic surface waters by a strong density gradient. Residence times of water in the sulfidic zone have been estimated previously as 1.5 to 7.5 years (Dahl et al., 2010). The lake is well-studied, with significant prior research on biogeochemical cycling and physical processes in the lake. Of particular importance to this study are the general geochemical and physical characterization (e.g. Del Don et al., 2001), Fe distributions and cycling in the water column (e.g. Berg et al., 2016; Ellwood et al., 2019), lake physical structure (Sommer et al., 2017; Sepúlveda Steiner et al., 2019; 2021), and the geological history and sediment geochemistry of the lake (e.g. Birch et al., 1996; Wirth et al., 2013; Berg et al., 2022). The lake has also been the focus of numerous prior geochemical studies as an analog system for conditions in the Proterozoic Ocean (e.g. Canfield et al., 2010; Dahl et al., 2010; Xiong et al., 2019; Ellwood et al., 2019).

Despite the elevated H_2S concentrations at depth in Lake Cadagno (up to ~10^2 μmol kg^{-1}), dissolved sulfate (~3 mmol kg^{-1}) and Fe concentrations are also high (>1 μmol kg^{-1}, Dahl et al., 2010; Ellwood et al., 2019). Surface sediments of Lake Cadagno are consistent with deposition in an anoxic environment, with elevated TOC and TS. Although deep waters are euxinic, surface sediments show high Fe_{HR}/Fe_{TOT} (0.76-0.88) and low Fe_{PYR}/Fe_{TOT} (0.16-0.30) (Xiong et al., 2019), consistent with records interpreted to reflect ferruginous conditions (e.g. Poulton & Canfield, 2011) and in good agreement with the bulk of the data from Proterozoic sediments (cf Farrell et al., 2021). In further agreement with ferruginous sediments deposited in the Proterozoic, surface sediment Mo/Al is less than 4.5 (cf. Dahl et al., 2010; Farrell et al., 2021), supporting the potential of Lake Cadagno as an analog system for understanding biogeochemical cycling in the Proterozoic Ocean.

S.1.2 Sampling and methodology

The Lake Cadagno water column was sampled on 28–29 August 2017 from a floating platform at the deepest part of the lake. Water samples (4 L) were collected with a peristaltic pump and filtered through 0.2 µm acid cleaned Supor filters (Acropak, Pall), and acidified to pH = 1.7 with HCl. Subsamples (0.5–2 L, yielding ~40–50 ng Cr) were spiked with a ^{50}Cr-^{54}Cr double spike and dried. Residues were refluxed with four aliquots of 5 mL 1 M HNO_3, each transferred to a 15 mL Teflon beaker and dried. To remove organic material, dried samples were refluxed overnight in 2 mL concentrated HNO_3 and 0.22 mL H_2O_2 (30% v/v). A white precipitate, which formed in some dried monimolimnion samples, was found not to contain significant amounts of Cr (see below) and was removed by centrifugation before column chromatography. Landsort Deep $\delta^{53}Cr$ samples were collected in the central Landsort Deep (site LD1; 435 m water depth; Häusler et al., 2018) onboard RV Poseidon (POS507, 29 October 2016) using the shipboard CTD and bottle rosette. The water samples were filtered through acid-cleaned 0.2 µm capsule filters into acid-cleaned PE bottles and acidified with HNO_3 (pH < 1.5). Samples were spiked and dried following the procedures described above. Landsort Deep samples were initially refluxed with 9:1 mixture of concentrated HNO_3 and 30% H_2O_2, dried, re-dissolved in 8 mL 7 M HNO_3 and microwave-digested. Reagents used in this study were either sub-boiling distilled in Teflon stills (acids) or Romil UpA and Fisher Chemical Optima grade (H_2O_2).

Pretreated water samples were then processed through three stages of column chromatography: (i) Fe removal using AG1-X8 resin with the sample in 6.4 M HCl (see also Scheiderich et al., 2015; Wei et al., 2018), followed by (ii) anion exchange chromatography using AG1-X8 resin with Cr oxidation followed by reductive elution and (iii) cation chromatography using AG50W-X8. Detailed descriptions of these procedures are provided in previous publications (e.g. Ball & Bassett, 2000; Yamakawa et al., 2009; Moos & Boyle, 2019; Rickli et al., 2019; Janssen et al., 2020; Nasemann et al., 2020). A small amount of rust-colored precipitate, likely Mn oxides (cf. Wei et al., 2018), formed in some deeper samples during the second chromatography step. This was removed by centrifugation. White and rust-colored precipitates were digested by refluxing in a mixture of 6.4 M HCl and 0.5 M HF, drying, and then dissolving in 6 mL 2 M HCl at 100° C. Samples were diluted to 1 M HCl and equilibrated at 80° C for at least one hour, then processed through cation exchange chromatography with AG50W-X8 resin (Yamakawa et al., 2009). Samples were analyzed by MC-ICP-MS and found to contain insignificant levels of Cr (< 1 ng, not shown).

Sediment traps were deployed on 10 July 2017 and recovered on 6 September 2017. Sample cups were rinsed with filtered surface water and then centrifuged, decanted to the extent that was possible without losing material, and stored in acid cleaned PP bottles and centrifuge tubes in the dark in a fridge until analysis. Given the poor settling nature of the sediment, some material may have been lost during trap recovery, centrifugation and decanting, and therefore sediment trap fluxes represent a minimum estimate of particulate fluxes. After evaporating to dryness, samples were microwave digested as with Landsort Deep water samples. Final calculated fluxes are corrected for the Cr and $\delta^{53}Cr$ of the surface water used to rinse the collection cups (<1 % total Cr in sediment trap samples), with uncertainty estimates derived from standard error propagation. Total Cr recovered in the sediment traps ranged from approximately 3000-7000 ng Cr (Table 1).

Lake Cadagno sediments were sampled using UWITEC Ltd. gravity and piston coring equipment in summer 2019 and summer 2020 (Berg et al., 2022). Piston coring down to a depth of ~940 cm

below the sediment water interface retrieved the entire lacustrine sediment record deposited since lake formation. Samples were freeze-dried and hand milled with an agate mortar and pestle. Near-total digests were prepared by weighing ~20 mg of sediment into pre-cleaned 14 ml Savillex Teflon beakers and adding 1.5ml of inverse aqua regia. Ten drops of 30% H_2O_2 were added to oxidize organic matter for 12 hours in the partially covered beaker. Samples were then refluxed at 140°C for 24 hours, dried and redissolved in 1 ml 0.5 M HNO_3. Authigenic sediment phases (organic matter, amorphous metal oxides and sulfides, see Figure 2, see also Berg et al., 2022) were targeted with a pH = 2 and 30% v/v H_2O_2 leach following the European Commission Standards, Measurement & Testing Programme (BCR) scheme of Rauret et al. (1999). Leach aliquots were dried and redissolved in 5 mL 0.5 M HNO_3. Subsamples from sediment leaches and digests were spiked with a ^{50}Cr-^{54}Cr double spike, dried and processed through the chromatographic column steps (i) & (iii) (see above). No lithogenic corrections were applied to the sediment leach data (see supplemental material for a detailed discussion).

Additional Lake Cadagno metal data were determined by Ion Chromatography at Eawag (dissolved Ca and Mg) following standard procedures or by ICP-MS at ETH Zurich (dissolved, leach and particulate Fe, Mn and Al; Vance et al., 2016). The Cadagno CTD dataset was obtained by performing bi-daily profiles using a Sea & Sun 75M CTD additionally equipped with pH, chlorophyll-a, dissolved oxygen, and turbidity sensors sampled at 8 Hz. Turbulence vertical microstructure profiles were obtained using a VMP-500 (RSI, Canada) free-falling at ~10 cm s^{-1} and sampling two fast FP07 thermistors (nominal time response of ~7 ms) at 512 Hz (Sepúlveda Steiner et al., 2019). Sediment TOC, TIC and TS were determined with a Carlo Erba EA 1108 Elemental Analyzer at the University of Bern following standard procedures. Landsort Deep [O_2] and turbidity were determined using a SBE 911plus CTD-rosette (Sea-Bird), and sampling and analysis for dissolved and particulate Fe and Mn are described in Dellwig et al. (2019). Total sulfide determinations followed procedures described in Cline (1969). In addition to the tables below and in the main text, chemical data are available at the following Zenodo dataset: Janssen et al. (2022).

S.2 Mixing in Lake Cadagno, August 2017

A storm event in August 2017, about 2.5 weeks before our dissolved sampling (28-29 August 2017) resulted in enhanced mixing above the chemocline. This may impact dissolved Cr profiles, especially in respect to the stability of [Cr] and $\delta^{53}Cr$ in upper waters.

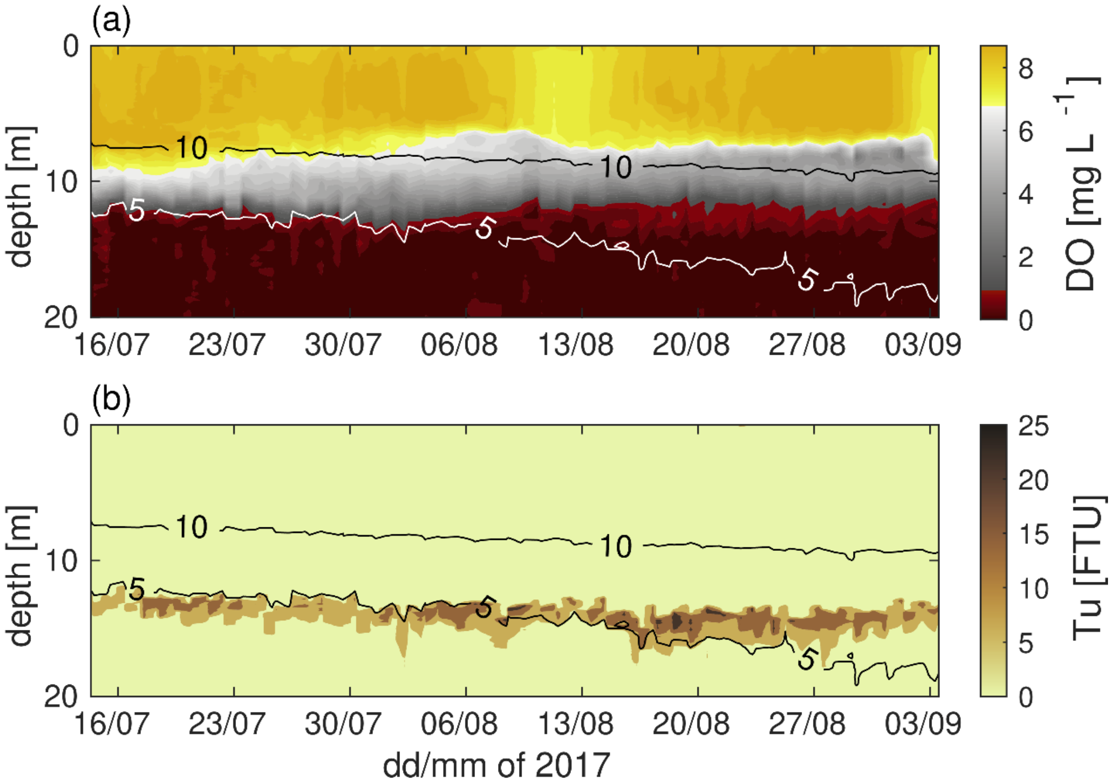

Figure S1: Dissolved oxygen (a) and turbidity (b) in Lake Cadagno during summer 2017. The 10° C and 5° C isotherms are shown as labelled contours. The turbidity peak reflects the microbial community found just below the chemocline. CTD data are available at the following Zenodo dataset: Sepúlveda Steiner, et al. (2022).

S.3 Chromium fluxes in Lake Cadagno

S.3.1 Sediment trap Cr fluxes and Cr inventories

A first order estimate of the time necessary to remove all dissolved Cr from the area between the 10 m and 14 m sediment trap was made using

- The measured pCr_{Auth} flux between these traps (Table 1, pCr_{Auth} at 14 m – pCr_{Auth} at 10 m = 4.3×10^3 ng Cr m^2 day^{-1})
- The water volume between these two traps, based on lake isobaths data (Table 2 in Del Don et al., 2001, treating these isobaths as stacked 1 m tall disks, and therefore maximizing the potential volume). Volume = 276,300 m^3

This dissolved Cr inventory was obtained by multiplying the water volume by the approximate Cr concentration (0.40 nmol kg^{-1}, and assuming a density of 1 kg l^{-1}), and dividing by the accumulation of particulate Cr between these traps, assuming this removal flux was acting only over the area at the bottom of this volume (therefore minimizing the total flux out). The result of this estimate is that all dissolved Cr would be removed from between 10 and 14 m depth in Lake Cadagno within 25 days, based on the calculated removal of particulate Cr, without comparable Cr resupply. Necessarily, based on the magnitude of molecular diffusive fluxes (K^{Cr}_{molec}, about four orders of magnitude lower than sediment trap fluxes), this resupply requires another physical transport process. In the deep waters of Lake Cadagno, this is turbulent diffusion.

S.3.2 Turbulent diffusivity and Cr transport

Measurements of microstructure-derived turbulent diffusivity (K_{oc}) were conducted over a 7-week period (Sepúlveda Steiner et al. 2019) spanning the course of our study and sediment trap deployments. These microstructure-derived turbulent quantities are snapshots of dynamic processes, which in total represent 78 distinct profiles determinations of instantaneous turbulent diffusivity. An effective diffusion rate is calculated following the diapycnal diffusivity model given in Osborn and Cox (1972). This model uses the smoothing rate of temperature variance, χ_θ (°C^2 s^{-1}) obtained from analyzing the vertical microstructure measurements (see Sepúlveda Steiner et al. 2019, 2021) by applying:

$$K_{oc} = \frac{\chi_\theta}{2\left(\frac{\partial \bar{T}}{\partial z}\right)^2}$$

In brief, the model incorporates turbulence stirring induced by external forcing (e.g., wind) into the diffusion rate estimates by considering temperature fluctuations and gradients as tracers. Considering

$$K_{eff} = K^{Cr}{}_{molec} + K_{OC}$$

this model constrains diffusivities for Cr. Microstructure diffusivities compare well (within a factor of 2) with those obtained from tracer release experiments. (e.g., Davis, 1994, Goudsmit et al., 1997). This agreement validates the method. For Lake Cadagno, Sepúlveda Steiner et al. (2019) reported excellent agreement between microstructure diffusivities and those obtained from a tracer release experiment (Wuest, 1994).

The intermittent or constantly varying nature of turbulent diffusivity is illustrated in Figure S2B. This significant natural variability indicates that, while both the 1st and 3rd quartile K_{oc} values are equally valid measured states, for a given unit of time, transport at the higher end of the range (e.g. 3rd quartile), results in significantly larger Cr flux than transport at the lower end (e.g. 1st quartile). Therefore, while the K_{oc} distributions and a median value are also shown in Figure S2B, the maximum likelihood estimation mean value (Baker & Gibson, 1987) is used for quantitative treatment, to better include the impact of elevated transport at the higher end of the observed K_{oc} range. The related statistics should not be interpreted as analytical uncertainties but as a signature of the natural variability of the system (turbulence in stratified fluids being intermittent). Finally, our results, clearly show that the system is dominated by turbulent diffusivity rather than molecular diffusivity in the surface and deep layers.

The determined turbulent diffusivity is then used to calculate expected diffusive Cr transport from [Cr]-enriched deep waters to the chemocline based on the observed [Cr] gradient.

$$F = K_{oc}\frac{\Delta Cr}{\Delta z}$$

with ΔCr and average K_{oc} taken from 13-14.5 m depth. The calculation is relatively insensitive to the exact range chosen, as $\frac{\Delta Cr}{\Delta z}$ is relatively uniform in Lake Cadagno deep water (ranging from 0.9×10^{-6} to 2.9×10^{-6} nmol cm^{-4}), with stronger Cr gradients closer to the chemocline (Figure 1C), and opposing K_{oc} variability with depth (Figure S2B). Given that these fluxes are estimated at the

order of magnitude level, taking into consideration heterogeneity of the system over the months sampled and uncertainty in isolating authigenic sediment trap fractions, such variability is insignificant.

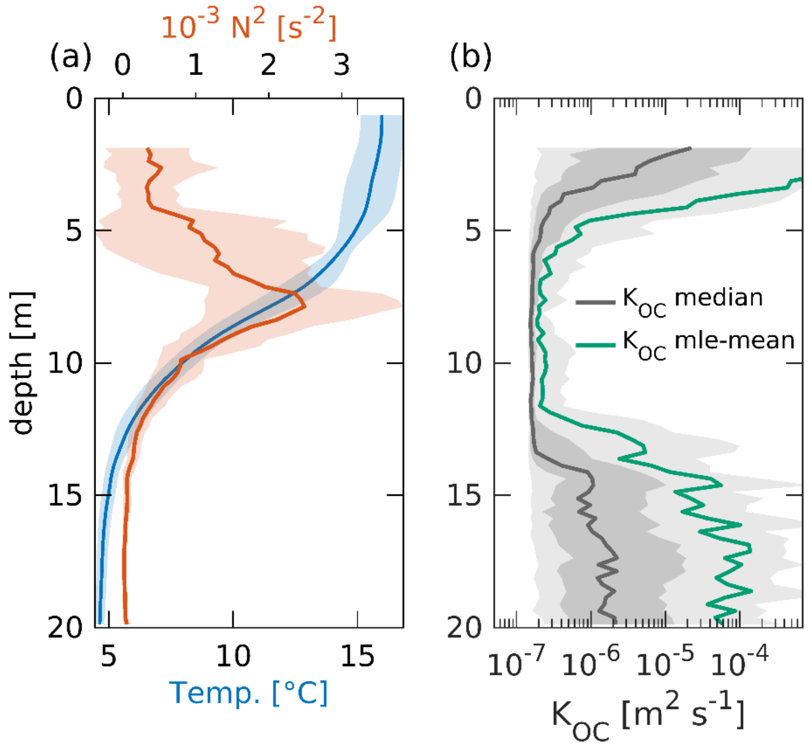

Figure S2: Turbulent diffusivity from July and August 2017.
Mean water column stability (red) and temperature (blue) are shown in (a) with shading representing 1 SD. Median turbulent diffusivities (black), with shading showing the range of 25% to 75% (dark grey) and 5% to 95% of the values (light grey), and MLE mean K_{oc} (green) are shown in (B).

S.3.3 Authigenic Cr burial flux

The burial flux of authigenic Cr in Lake Cadagno surface sediments is estimated based on previously published sediment accumulation rates (4-6 mm yr^{-1}, Birch et al., 1996), measured sediment densities and porosity (Table S6) and the authigenic Cr content of surface sediments (~8 ppm, Table S4) following:

$$F_{Burial} = \text{Sediment Accumulation Rate} \times \rho \times (1-\beta) \times Cr_{Auth}.$$

The resulting flux (49-74 ng cm^{-2} yr^{-1}, or 1.3-2.0 × 10^3 nmol m^{-2} d^{-1}) is approximately 25% of the estimated sinking particulate and upward diffusive fluxes near the chemocline and approximately 50% of the sinking particulate flux from the deepest sediment trap. This supports the release of a significant fraction of Cr from particulates into deep waters during particle sinking and at the sediment surface. This is also consistent with (elevated) dissolved and (lowered) particulate Cr observed at depth.

Depth	Cr	δ^{53}Cr	2SEM	Note	Ca	Mg	Fe	Mn	Al	P
m	nmol kg^{-1}	‰	‰		µM	µM	µmol kg^{-1}	µmol kg^{-1}	nmol kg^{-1}	µmol kg^{-1}
1	NA	NA	NA	NA	466	292	0.09	0.03	188.8	0.04
2	0.497	0.86	0.03	NA	470	299	0.09	0.03	181.5	0.06
3	NA	NA	NA	NA	467	301	0.09	0.03	188.2	0.09
4	0.489	0.85	0.03	NA	468	300	0.09	0.03	175.7	0.09
5	NA	NA	NA	NA	470	297	0.11	0.03	173.0	0.06
6	NA	NA	NA	NA	469	295	0.13	0.04	162.3	0.06
7	0.435	0.77	0.04	NA	540	326	0.14	0.26	102.0	0.09
8	0.406	0.72	0.04	NA	659	399	0.15	0.36	60.1	0.13
9	0.384	0.53	0.03	NA	793	490	0.12	0.45	35.1	0.14
10	0.385	0.57	0.05	NA	851	531	0.12	0.61	29.0	0.16
11	0.405	0.50	0.03	NA	960	607	0.14	1.29	18.1	0.19
12	0.426	0.40	0.04	NA	980	627	0.10	1.92	20.0	0.18
13	0.470	0.34	0.04	NA	1025	656	0.07	2.47	14.2	0.14
13.5	0.610	0.40	0.04	†	1026	662	0.87	2.72	11.7	0.15
14	0.662	0.37	0.03	†	1038	663	1.24	2.73	14.6	0.21
14.5	0.670	0.34	0.03	†	1041	673	1.32	2.71	14.8	0.22
15	0.785	0.28	0.03	†	1049	668	1.42	2.71	15.1	0.27
15.5	0.809	0.43	0.03	†	1046	667	1.61	2.64	18.1	0.27
16	0.803	0.38	0.03	†	1052	679	1.63	2.69	23.8	0.38
17	0.831	0.42	0.02	‡	1047	672	1.61	2.69	17.9	0.41
18	0.892	0.45	0.04	†	1080	695	1.66	2.68	21.6	0.87

Table S1: Water column data. Ca and Mg were determined by ion chromatography, while Fe, Mn, Al and P were determined by ICP-MS. δ^{53}Cr uncertainties are internal 2SEM, from the analysis. External reproducibilities are typically around ± 0.02 to ± 0.03 ‰ (2SD) for NIST standards, and have been previously determined as ± 0.033 ‰ based on full sample replicates (Janssen et al., 2020). NA indicates data not available or no note. †δ^{53}Cr represents a weighted mean from replicate analysis of a single processed sample. ‡δ^{53}Cr represents a weighted mean from replicate analysis of two independently spiked and processed sample aliquots.

S.4: Particulate oxide shuttles and groundwater

By comparing Cr, Fe, P, Mn and Mg distributions in Lake Cadagno, Figures S3 and S4 look at the roles of vertically acting processes (mixing, particulate fluxes) and groundwater sources in shaping Cr distributions in Lake Cadagno.

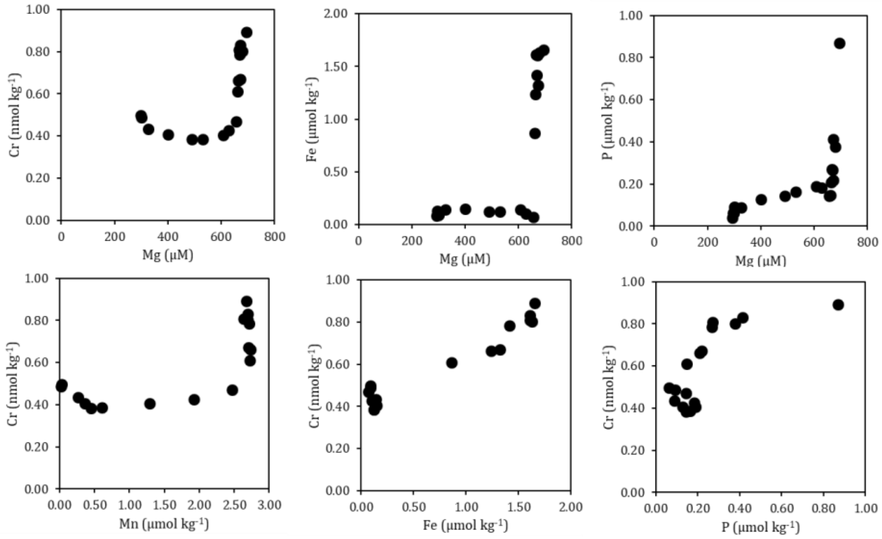

Figure S3: Chromium, Fe, P, Mn and Mg bi-variant plots. Chromium, Fe and P distributions are compared to Mg (top), and Cr is compared to Mn, Fe, and P (bottom). The nonlinear distributions of Cr, Fe and P relative to Mg indicate removal at intermediate depth (intermediate Mg), with significant accumulation in deep waters at relatively stable and high [Mg]. The broadly similar Cr, Fe and P, showing enrichment at depth, are consistent with reduction and dissolution of metal oxides and the overall importance of an Fe redox shuttle, while differences between Mg and Cr, Fe and P distributions indicate Cr, Fe and P distributions are not consistent with the primary control of a groundwater source. Chromium correlates better with Fe than with Mn, indicating that Mn oxides may be less important than Fe oxides in this redox shuttle.

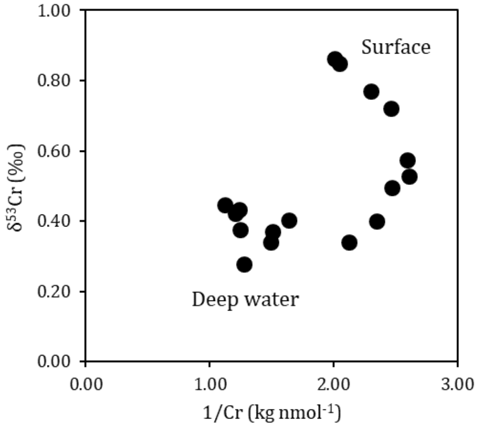

Figure S4: Cr-δ^{53}Cr plot. Zones within the Lake Cadagno water column are labeled. Chromium is removed at intermediate depth (δ^{53}Cr ≈ 0.6 ‰) and isotopically light Cr accumulates below this, with mixing processes above and below this removal zone. Isotopically heavy deep water may reflect an isotopically heavy groundwater source.

Depth	Al	Mn	Fe	No Detrital Correction			Detrital Correction		
m	µg m^{-2} day^{-1}	µg m^{-2} day^{-1}	µg m^{-2} day^{-1}	ng Cr m^{-2} day^{-1}	δ^{53}Cr	2SEM	ng Cr m^{-2} day^{-1}	δ^{53}Cr	2SEM
10	1609	627.7	2810	4236	-0.07	0.03	2401	-0.03	0.09
14	2343	82.2	5068	9231	-0.05	0.03	6701	-0.02	0.05
20	3234	43.4	4899	7205	-0.02	0.03	3647	0.09	0.11

Table S2: Sediment trap data. Chromium data are presented with and without detrital corrections, based on measured Al and a detrital Cr/Al of 0.00113 (from a Cr content of 92 ppm and an Al content of 8.15 % by mass (15.4 % Al_2O_3 by mass); Rudnick & Gao, 2014). As discussed in Section S.5, the corrected values are likely over-corrected, and the true authigenic Cr fluxes and isotopic composition likely lie between the uncorrected and corrected values.

Standard	Cr	δ^{53}Cr	2SEM
	ppm	‰	‰
SGR-1b – Near Total Digest	31.7	0.31	0.02
SGR-1b – Zhu et al., 2018	32.30	0.29	0.03
SGR-1b – Wu et al., 2020	NA	0.30	0.02
SGR-1b Leach 1	13.87	0.32	0.03
SGR-1b Leach 2	15.04	0.25	0.04
SDO-1 – Near-Total Digest	NA	-0.11	0.02
SDO-1 – Schoenberg et al., 2008	53.8	-0.08	0.05
SDO-1 – Zhu et al., 2018	55	-0.09	0.03

Table S3: Sediment standards. Near-Total digests and sediment leaches were conducted on the standard reference materials SGR-1b and SDO-1, with values determined in this study compared to literature data. The subsample of SDO-1 had been taken from an aliquot that was previously ashed, precluding an accurate determination of actual initial sample mass, which must be more than the weighed subsample. The Cr concentration obtained (60.4 ppm) is thus not useful as an external calibration value. The concentration is slightly higher than the reference concentration (~10%), as expected for the specific sample treatment.

Sample	Depth	Cr	$\delta^{53}Cr$	2SEM	Al	Fe	Mn	Cr (Detrital-Corrected) (Al-based)				TOC	TS	TN	C/N	Si	P
	cm	ppm	‰	‰	%	%	ppm	**ppm**	**% total**	**$\delta^{53}Cr$**	**2SEM**	**(wt %)**	**(wt %)**	**(wt %)**		**ppm**	**ppm**
Cad20 0-1	0.5	8.0	0.11	0.03	0.24	0.96	51.7	5.22	66	0.24	0.07	17.6	1.8	2.2	7.9	324	714
Cad20 1-2	1.5	8.4	0.13	0.03	0.22	1.06	41.3	5.92	70	0.24	0.06	18.2	2.0	2.2	8.1	200	526
Cad20 2-3	2.5	7.4	0.09	0.02	0.22	0.91	49.0	4.97	67	0.20	0.06	15.1	1.7	1.7	8.7	162	416
Cad20 3-4	3.5	7.5	0.05	0.03	0.22	0.94	38.8	5.05	67	0.13	0.07	14.5	1.8	1.7	8.3	236	359
Cad20 4-5	4.5	7.6	0.02	0.02	0.21	0.96	33.0	5.22	69	0.09	0.05	14.8	1.9	1.7	8.6	168	257
Cad20 5-6	5.5	7.4	0.00	0.02	0.22	0.96	32.3	4.85	66	0.06	0.06	13.5	1.9	1.5	8.9	145	180
Cad20 6-7	6.5	9.5	-0.01	0.03	0.26	1.15	44.9	6.53	69	0.04	0.06	14.3	2.1	1.6	9.1	324	223
Cad20 7-8	7.5	9.0	-0.05	0.03	0.26	1.27	67.6	6.05	68	-0.01	0.07	15.8	2.5	1.8	8.9	187	161
Cad20 8-9	8.5	10.3	-0.10	0.03	0.29	1.42	72.8	7.05	69	-0.08	0.06	12.8	2.6	1.4	9.3	151	161
Cad20 9-10	9.5	12.0	-0.10	0.02	0.34	1.45	105	8.14	68	-0.08	0.06	12.4	2.8	1.3	9.7	187	180
Cad20 20-21	20.5	2.6	-0.04	0.04	0.21	0.88	425	0.26	10	0.77	1.01	1.5	1.0	0.2	6.7	91	99
CAD19 PORE 200-300	227	6.2	0.01	0.04	0.31	1.50	1051	2.61	42	0.18	0.17	NA	NA	NA	NA	166	182
CAD19 PORE 400-500	440	6.1	0.06	0.03	0.24	1.21	528	3.41	56	0.21	0.10	NA	NA	NA	NA	95	73
CAD19 PORE 700-800	724.5	9.9	0.04	0.03	0.38	2.19	1135	5.60	56	0.17	0.10	NA	NA	NA	NA	643	231

Table S4: Sediment leaches.

Sample	Digest	Depth	Cr	$\delta^{53}Cr$	2SEM
	Set	cm	ppm	‰	‰
Cad20 0-1	2	0.5	43.0	-0.05	0.02
Cad20 1-2	2	1.5	42.3	-0.03	0.02
Cad20 2-3	2	2.5	40.8	-0.05	0.02
Cad20 3-4	2	3.5	43.9	-0.13	0.02
Cad20 4-5	2	4.5	45.6	-0.08	0.02
Cad20 5-6	2	5.5	49.2	-0.08	0.03
Cad20 6-7	2	6.5	50.0	-0.12	0.02
Cad20 7-8	2	7.5	48.8	-0.15	0.03
Cad20 8-9	2	8.5	51.7	-0.13	0.03
Cad20 9-10	2	9.5	55.4	-0.13	0.02
Cad20 20-21	2	20.5	74.6	-0.19	0.02
PORE 100-200 80-81	1	171.5	73.4	-0.16	0.04
PORE 100-200 87-89	1	180	80.3	-0.18	0.03
PORE 200-300 24-25	1	227	66.9	-0.11	0.03
PORE 200-300 24-25	2	227	71.1	-0.13	0.02
PORE 200-300 31-33	1	236	86.3	-0.13	0.02
PORE 300-384 46-49	1	359	58.2	-0.15	0.02
PORE 300-384 70-72	1	383	62.0	-0.13	0.04
PORE 400-500 65-66	1	440	67.8	-0.11	0.04
PORE 400-500 65-66	2	440	68.3	-0.10	0.02
PORE 400-500 82-83	1	456	67.3	-0.14	0.03
PORE 500-600 59-61	1	531.5	66.5	-0.18	0.02
PORE 500-600 71-72	1	542.5	73.5	-0.16	0.03
PORE 600-684 17-19	1	589	68.0	-0.14	0.02
PORE 600-684 17-19	2	589	75.5	-0.15	0.02
PORE 600-684 26-27	1	598.5	75.8	-0.11	0.02
PORE 600-684 69-70	1	644	70.8	-0.15	0.03
PORE 600-684 78-79	1	652.5	75.5	-0.13	0.02
PORE 600-684 78-79	2	652.5	75.6	-0.13	0.02
PORE 700-800 17-18	1	683.5	85.5	-0.14	0.03
PORE 700-800 34-36	1	698.5	78.4	-0.26	0.04
PORE 700-800 58-59	1	723.5	69.9	-0.08	0.03
PORE 700-800 76-78	1	724.5	65.3	-0.11	0.03
PORE 700-800 76-78	2	724.5	78.8	-0.13	0.02
PORE 800-900 8-10	1	769.5	71.5	-0.12	0.03
PORE 800-900 25-27	1	786.5	62.4	-0.14	0.03

Table S5: Sediment near-total digests. Replicate preparation and analysis of sediment samples suggests the external reproducibility for [Cr] is around 10% (likely due to weighing error on small sample sizes, along with potential sample heterogeneity); however, no differences were observed in stable isotope composition among replicates.

Depth	Mass wet	Mass dry	H_2O mass
cm	g	g	g
0	14.7627	9.7040	5.0587
1	14.8089	9.8197	4.9892
2	15.1788	9.7802	5.3986
3	14.6130	9.6861	4.9269
4	14.3622	9.8144	4.5478
6	14.5668	9.8361	4.7307
8	14.8232	9.9446	4.8786
10	14.4063	9.9514	4.4549

Table S6. Wet and dry sediment mass. Masses are for 5 mL sample volumes for samples from the 2019 coring campaign. Average values from the upper 10 cm were used for Cr burial flux calculations in Table 1 (see also Section S.3.3).

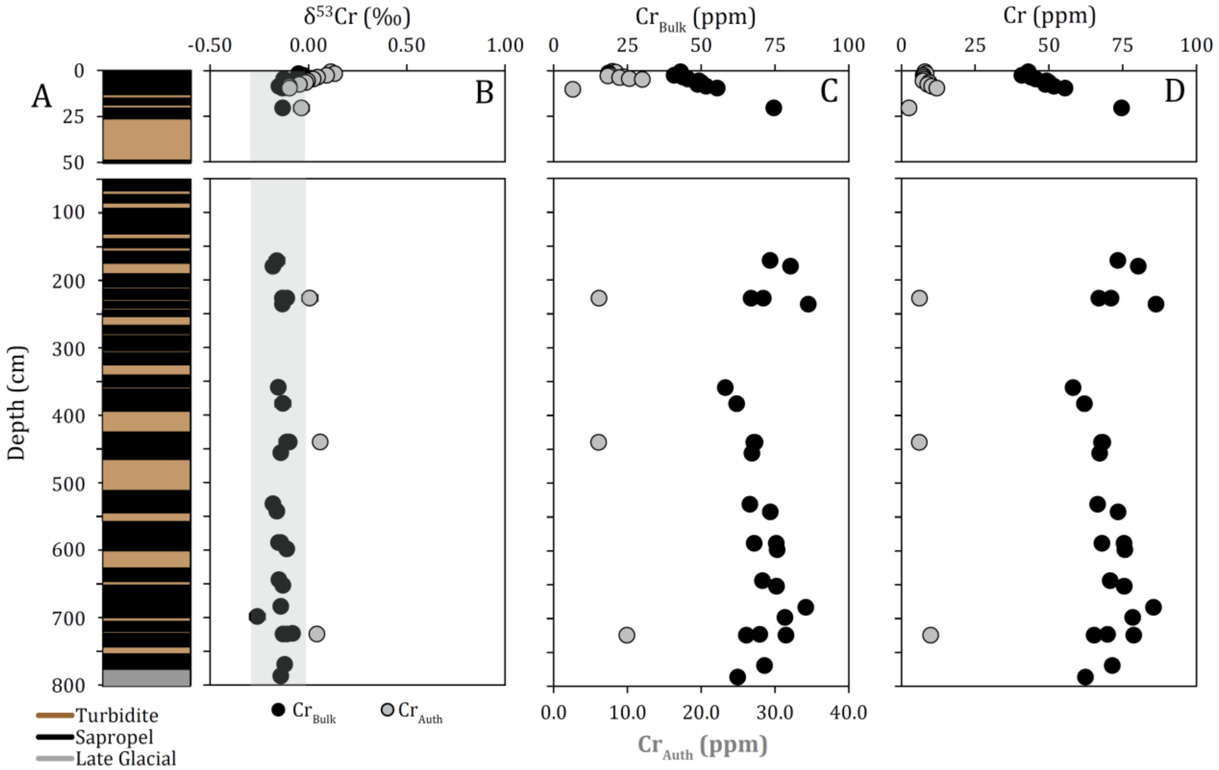

Figure S5: Full sediment core [Cr] and δ^{53}Cr data from Lake Cadagno. Bulk sediment core composition (Panel A) is shown with δ^{53}Cr (Panel B) and [Cr] (Panels C & D). Near-total digest data (Cr_{Bulk}) are shown in black and leaches (Cr_{Auth}) in grey. The vertical scaling is expanded in the upper 50 cm to show trends in upper sediment samples. The grey box in Panel B indicates average detrital δ^{53}Cr (Schoenberg et al., 2008). Note the different scaling of Cr Leach (Cr_{Auth}) and Near-Total Digest (Cr_{Bulk}) in Panel C. Panel D shows the two at the same scaling.

S.5 Discussion of authigenic corrections

In sediments and sinking particles in natural aqueous systems, Cr will be present in authigenic as well as detrital phases. However, it is only the authigenic phases that might provide insight into in situ Cr cycling and yield potential paleoproxy information. The two main and often combined strategies to isolate the authigenic signal from the composite sample matrix are chemical leaches designed to target various more labile phases, and corrections based on immobile silicate-hosted elements (i.e. Al and Ti) (Equation S1, where UCC indicates average Upper Continental Crust, e.g. Rudnick & Gao, 2014).

$$Cr_{Authigenic} = Cr_{Measured} - Al_{Measured} \times \frac{Cr_{UCC}}{Al_{UCC}} \qquad \text{(Equation S1)}$$

However, both of these approaches are prone to error. First, while leaches are designed to target specific authigenic phases, these leaches are operationally defined and attack multiple authigenic and detrital phases with varying strength (e.g. Rauret et al., 1999; Frank et al., 2019). Second, detrital corrections rely on a well-constrained ratio of Cr to the chosen normalizing detrital element, which, however, is known to vary in crustal material (e.g. Cole et al., 2017). Furthermore, such corrections assume that detrital sources strongly dominate the extracted signal of the chosen normalizing element, with negligible contributions from authigenic phases. Therefore, there is no perfect isolation of authigenic signals, and normalization approaches must be considered carefully based on the environmental conditions and available data of each scenario.

We present near-total sediment digests, sediment leaches targeting organic matter and sulfides (30% w/w H_2O_2 at pH = 2 with HNO_3, Rauret et al., 1999), and aggressive oxidizing digestions of sediment trap material (refluxing with a mixture of 90% v/v concentrated HNO_3 + 10% v/v 30% w/w H_2O_2 followed by microwave digests in 7 M HNO_3). Because near-total sediment digests were performed to characterize the bulk sediment Cr signal, no corrections were made to these data. Previous research in black shales has shown that the lability of Al in detrital phases is more comparable to Cr than that of Ti (Frank et al., 2019). Therefore, we base corrections on Al and, regarding whether to apply Al-based detrital corrections to our sediment leaches and sediment trap digestions, we consider our leaching/digestion protocol and ancillary metal data.

Sediment trap samples (denoted as pCr in the text and figures) included poorly settling material. Therefore they were stored with some overlying supernatant present, which was eventually evaporated on a hot plate (due to elevated Cr filter blanks, e.g. Scheiderich et al., 2015, we avoided collecting particles on filters followed by filter digestion). Consequently, it was not clear that native speciation was preserved and gentle digestions were thus not applied. Instead, an approach with HNO_3 and H_2O_2 was chosen for aggressive oxidation of the organic-rich samples and dissolution of FeMn oxides, while minimizing leaching of aluminosilicates. This more aggressive approach likely liberated some detrital Cr, and a correction is applied using average Cr/Al for upper continental crust (denoted as pCr_{Auth} in the text and figures, Rudnick & Gao, 2014), and the δ^{53}Cr of igneous material (δ^{53}Cr = 0.12 ± 0.10, 2SD, Schoenberg et al., 2008). However, we note that dissolved [Al] in Lake Cadagno is clearly non-conservative, and instead reflects the formation of non-detrital Al phases (Figure S6, see also Ellwood et al., 2019). Therefore, we caution that this correction likely overestimates the detrital contributions and therefore leads to an underestimate of the true authigenic Cr flux. Similarly, the correction likely

overcorrects $\delta^{53}Cr$ to excessively high authigenic values. The true authigenic fluxes and isotope compositions likely lie between the corrected and the uncorrected ones. However, given that all corrected $\delta^{53}Cr$ values are within uncertainty of uncorrected values, this does not impact our interpretations. Both uncorrected and corrected data are shown in Table 1 and Figures 1 & 2.

For our sediment samples, oxidative leaches were applied to sediment subsamples (25-35 mg) in 2 mL 30% H_2O_2 in low molarity acid (0.01 M HNO_3, pH = 2), designed to target authigenic phases. This procedure was chosen because it targets the main authigenic phases present in our samples: organic matter (≥ 14% TOC in most of our sediment samples), sulphides (1-3% TS, Figure 2 & Table S4) (e.g. Rauret et al., 1999). In addition, our sediments may contain potential fast-sinking amorphous metal oxides that survive reduction before reaching the sediment surface (Berg et al., 2022). Acidic H_2O_2 efficiently dissolves Mn oxides (e.g. Neaman et al., 2004). While H_2O_2 is known to react catalytically with rather than dissolve Fe oxides (e.g. Kwan & Voelker, 2002), and is only poorly effective at dissolving crystalline Fe oxides (Neaman et al., 2004), the acidic environment of our leach is capable of dissolving low levels of amorphous Fe oxides such as ferrihydrite (Shi et al., 2011). Given a sediment surface Fe(III) of approximately 100 µmol Fe g^{-1} dry sediment (Berg et al., 2022), and about 25-35 mg sediment in a 2 mL leach, this would yield dissolved Fe(III) from amorphous oxides up to ~1 mM, much lower than the dissolvable Fe(III) in this leach solution based on previous studies (e.g Shi et al., 2011). Therefore, the leach used should also effectively target authigenic Cr in the low levels of metal oxides that may reach the sediment surface at Lake Cadagno.

The weakly acidic nature of this leach, which is one to two orders of magnitude lower molarity than previous acidic leaches applied to similar sediments (e.g. Reinhard et al., 2014; Frank et al., 2019), is unlikely to strongly attack silicates. Indeed our leachate Al data do not appear to reflect extraction of detrital phases. Rather, leachate Al shows positive correlations with other elements known to have major authigenic phases in sediments (TS, Mn, Fe) and no correlation with leachate Si hosted, for instance, in clays (Figure S7). Furthermore, we find the lowest leachate Al in the sample with by far the largest detrital composition (20.5 cm, >90% detrital, in comparison with <60% to a maximum of ~70% detrital in other samples). Therefore, we do not apply any detrital corrections to our sediment leachate data. However, for the sake of comparison, Table S4 shows the calculated values assuming all of the leached Al were detrital. Again, for the reasons outlined above, we argue this is not correct and we stress that we believe uncorrected values to more accurately reflect authigenic Cr than these recalculated values. Nevertheless, the qualitative results remain the same regardless of which data are used (Figure S8). Namely, these are:

- $[Cr]_{Auth}$ increases with depth
- $\delta^{53}Cr_{Auth}$ decreases with depth
- $\delta^{53}Cr_{Auth}$ is distinct from the water column, with a variable offset depending on sediment depth and water column depth (i.e. euxinic zone average or Cr removal zone).

Indeed, applying Al-based detrital corrections magnifies the downcore features. The interpretations are thus supported by 'corrected' and uncorrected data, despite clear artefacts with the 'corrected' data.

For future studies, it is important to consider these results in the context of detrital corrections using normalizing elements. Starting from the potential endmembers of:

1. A 100% digestion of sediments (whereby elements like Al will overwhelmingly show detrital control), and
2. A hypothetical perfect leach, releasing only authigenic fractions (whereby Al will entirely reflect authigenic phases)

one should carefully evaluate where the chosen leach and depositional environment may fall. This should include:

1. Estimates of the degree to which the normalizing element behaves non-conservatively (and therefore how much non-detrital signal may be expected)
2. The relative magnitude of non-conservative behavior of the normalizing element and the element of interest (i.e. potential Al_{Auth}/Cr_{Auth}). In the case of Lake Cadagno, dissolved Al shows variability on the order of 100 µg kg^{-1}, while Cr is on the order of 30 ng kg^{-1}, resulting in a high potential Al_{Auth}/Cr_{Auth}.
3. How well the leach is believed to isolate authigenic features, and therefore the potential degree of detrital contamination.

While there is no perfect solution for authigenic corrections, one must ensure that applying such a correction does not introduce more artefacts than it may correct. As leaching protocols are refined and become progressively more gentle and better extract authigenic phases while limiting detrital phase extraction, it is increasingly likely that authigenic corrections following standard elemental ratio approaches (e.g. Equation S1) may do more harm than good.

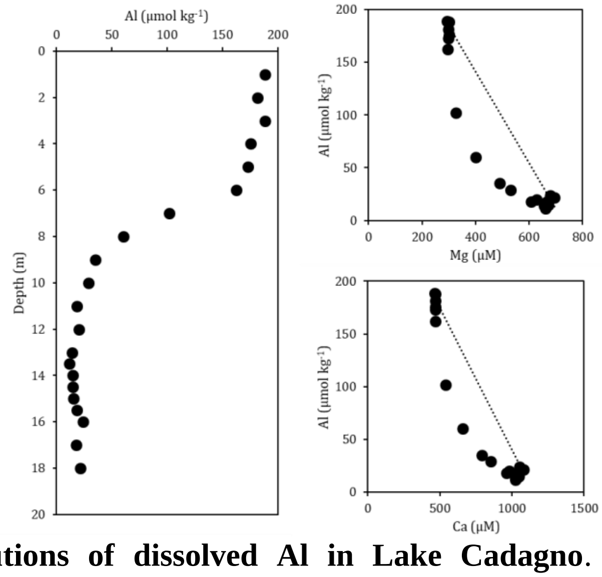

Figure S6: Distributions of dissolved Al in Lake Cadagno. Depth profiles and comparisons to major cations (Ca, Mg) show the non-conservative behavior of Al in Lake Cadagno. The mid-depth [Al] minimum and mixing diagrams, where [Al] falls below a mixing line between surface and deep endmembers, reflect the mid-depth removal of Al to authigenic phases.

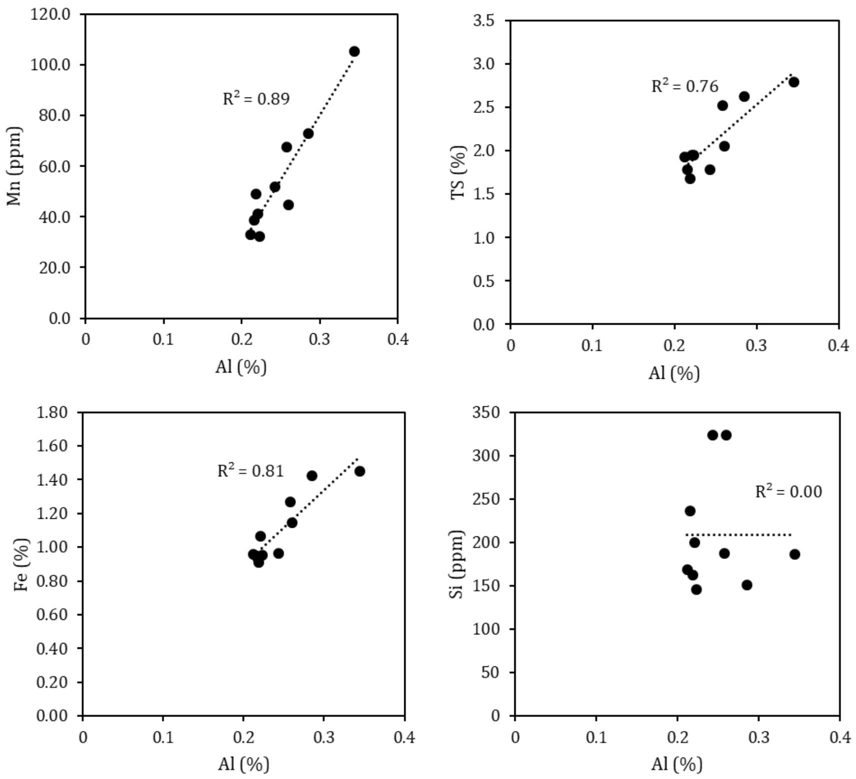

Figure S7: Relationships between Al and other parameters in short core (0-10 cm) sediment leaches.

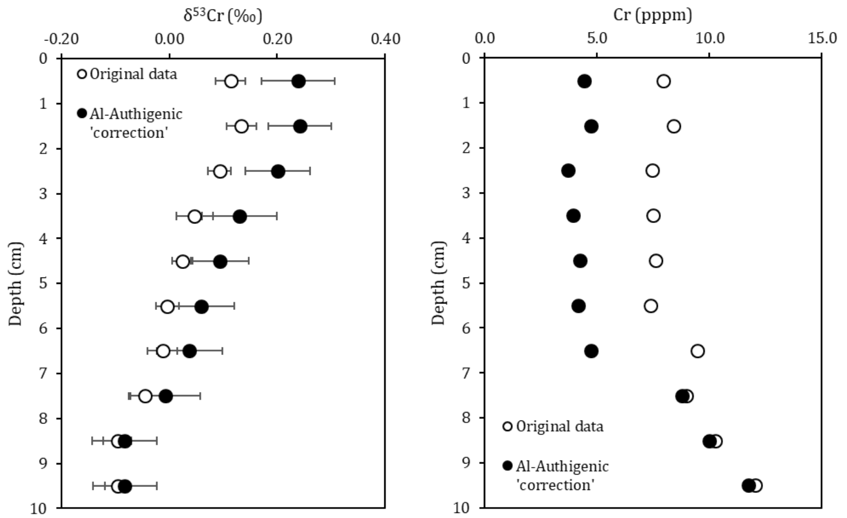

Figure S8: Comparison of original sediment leachate and detrital 'corrected' data based on Al. Trends are the same for both the original data and data with detrital 'corrections' based on Al. For δ^{53}Cr all data are indistinguishable within uncertainty below the 2.5 cm sample. As discussed in section S.5, the Al-based 'corrections' create clear artefacts due to non-trivial authigenic Al phases, and these data therefore do not represent true authigenic Cr distributions. Rather, the original data are believed to more accurately reflect authigenic Cr.

S.6 Landsort Deep (Baltic Sea) data

Baltic Sea samples for Cr were collected on 29-Oct-2019 in the central Landsort Deep (site LD1, 58° 38.4460 N, 18° 15.9343 E; 435 m water depth, see also Häusler et al., 2018), with higher resolution sampling for Fe and Mn conducted at the same site 4 days prior.

**Depth**	**Cr**	**δ^{53}Cr**	**2SEM**	**Yield**	H_2S	dMn	dFe	pMn	pFe
**m**	**nmol kg^{-1}**	**‰**	**‰**	**%**	µM	µM	µM	µM	µM
39.7	1.06	0.56	0.03	56.4	0.0	0.0	0.01	0.004	0.007
79.5	0.75	0.38	0.05	59.9	0.0	2.3	0.01	0.940	0.052
88.3	1.15	0.23	0.03	57.4	2.3	4.4	0.04	0.014	0.019
119.1	1.15	0.29	0.03	61.1	5.3	4.5	0.25	0.005	0.009
300.6	1.17	0.29	0.04	58.3	18.6	2.8	0.24	0.001	0.005
429.3	1.64	0.18	0.03	52.0	17.7	2.6	0.24	0.001	0.005

Table S7: Landsort Deep (Baltic Sea) water column Cr data.

**Depth**	**dMn**	**pMn**	**dFe**	**pFe**
m	µM	µM	µM	µM
5	0.02	0.012	0.06	0.024
50	0.01	0.029	0.03	0.021
55	0.04	0.042	0.03	0.022
60	0.01	0.155	0.01	0.023
65	0.01	0.223	0.02	0.026
70	0.01	0.265	0.01	0.035
75	0.01	0.465	0.02	0.063
80	0.01	0.399	0.02	0.043
83	0.01	0.365	0.01	0.034
86	0.01	0.868	0.02	0.072
89	0.66	1.353	0.03	0.072
91	2.42	1.175	0.03	0.103
93	4.16	0.746	0.07	0.143
98	5.68	0.001	0.47	0.077
105	4.67	0.001	0.56	0.057
110	4.21	0.001	0.43	0.049
115	3.97	0.002	0.40	0.046
120	3.74	0.001	0.40	0.035
130	3.51	0.001	0.39	0.019
150	3.15	0.001	0.37	0.015
180	2.65	0.000	0.34	0.008
250	2.36	0.001	0.29	0.004
340	2.27	0.001	0.28	0.019
380	2.27	0.000	0.28	0.015
420	2.26	0.000	0.27	0.012
431	2.30	0.001	0.27	0.013

Table S8: Landsort Deep (Baltic Sea) water column Fe and Mn data.

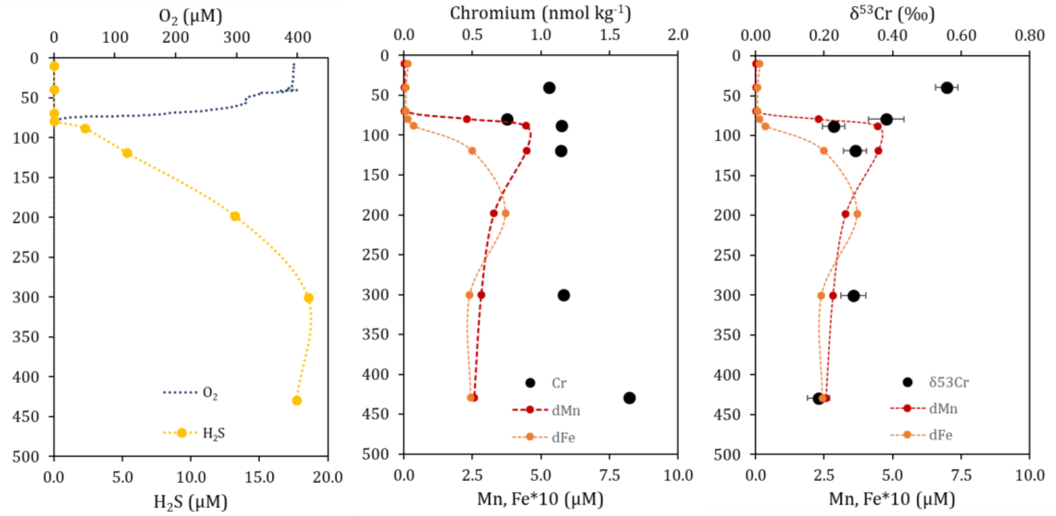

Figure S9: Landsort Deep (Baltic Sea) water column profiles. Note that Fe is scaled by a factor of 10 to fit on the same axis as Mn.

S.7 Compilation of literature data

Literature data from redox stratified basins are compiled from Saanich Inlet (Emerson et al., 1979, BC Canada, see also Davidson et al., 2020), the Black sea (Cr: Mugo, 1997; Fe & Mn: Lewis & Landing, 1991; S: Luther III et al., 1991), Esthwaite water (UK, metals: Achterberg et al., 1997; S: Sholkovitz & Copland, 1982) and Hall Lake (Wa USA; Ballistrieri et al., 1994) for Figure 3. The table below shows ranges of H_2S, dissolved Fe and Fe/H_2S (rounded to the nearest order of magnitude) in anoxic deep waters at these sites from the above sources, as well as Lake Cadagno and Landsort Deep site (Baltic Sea) (this study, Lake Cadagno H_2S data from Ellwood et al., 2019).

In addition to the ferruginous Hall Lake, data from the ferruginous Lake Matano are also available (Crowe et al., 2008) but have not been included here. Lake Matano site shows broadly similar behavior (incomplete Cr removal at the chemocline, and increasing Cr below the chemocline relative to chemocline values); however, the ultramafic setting results in Cr concentrations (>100 nM) considerably higher than other modern lakes, inland seas and oceans (~0.5-6 nM).

Site	Fe (μM)	H_2S (μM)	Fe/H_2S
Lake Cadagno	0.1-1.7	7-120	10^{-2}-10^{-1}
Landsort Deep (Baltic Sea)	0.04-0.4	2-19	10^{-2}
Black Sea	0.01-0.3	0.1-400	10^{-4}-10^{-2}
Saanich Inlet	0.4-0.9	4-21	10^{-2}-10^{-1}
Esthwaite Water	2-115	1-4	10^{0}-10^{1}
Hall Lake	10-750	1-10	10^{1}-10^{3}

Table S9: Sulfide, Fe and Fe/H_2S ratios in redox-stratified systems included in Figure 3.

References

For references cited in the supplemental material, see main text.
